# Succession and proliferation of opportunistic pathogens on leafy greens at retail markets

**DOI:** 10.1186/s12866-026-04823-0

**Published:** 2026-03-07

**Authors:** Dineo Attela Mohapi, Sebolelo Jane Nkhebenyane

**Affiliations:** Department of Life Science, Centre for Applied Food Safety and Biotechnology, Central University of Technology, Free State, Private Bag X20539, Bloemfontein, 9301 South Africa

**Keywords:** Contamination, Cold-chain, Hygiene, Leafy greens, Proliferation

## Abstract

Leafy greens consumed raw are consider potential reservoirs for opportunistic pathogens and are able to cross geographical barriers by attaching to natural apertures and thrive in new niche. This study aimed to enumerate microbiota and identify microbial species isolated from spinach (Spinacia oleracea L.) and cabbage (Brassica oleracea var. capitata L.) at markets that are mainly supplied by small-scale farms in the Free State, South Africa. Small-scale farm pre-harvest and post-harvest practises contribute to succession and proliferation of pathogens at markets. Analytical profile index (API) tests were conducted for final confirmation and identification to a specie level. Among leafy green samples enumerated, 17.4% and 13.9% were contaminated by E. coli which is highly prevalent in cabbage and spinach followed 13.1% and 13% P. mirabilis and 12.65 and 9.6% C. cloacae including 4.4% of L. monocytogenes and 4.9% L. ivanovii in spinach. Listeria species was not detected in any cabbage samples. The highest count of Enterobacteriaceae ranged from 1–4.9 log CFU/g, aerobic mesophilic bacteria ranged from 2–4.9 log CFU/g in spinach followed by aerobic mesophilic with 2–3.9 log CFU/g and Enterobacteriaceae range from 2–4.9 log CFU/g and aerobic mesophilic while the minimum was in spinach samples 1–2.9 log CFU/g for Listeria species. Agronomic parameters in small-scale farms, including the cold chain abruption, contribute to the succession of opportunistic microorganisms as they are not subjected to any lethal process employed to effectively kill pathogenic organisms. The data is crucial for evaluating the pathogenic potential trend and its aetiology regarding cold chain abruption cross-contamination, and proliferation due to opportunistic pathogenic microorganisms at markets.

**Supplementary Information:**

The online version contains supplementary material available at 10.1186/s12866-026-04823-0.

## Introduction

Consumers perceive leafy green vegetables from retail markets as convenient and also as an essential part containing variety of imperative sources to human health. South Africa accounts for 2.6 million tons which is 92.5% of fresh vegetables and only 4% is exported [1]. From 2019 to 2020, the horticultural industry was highlighted as the second-largest agricultural sector following livestock production worth R141 billion and it contributed over R 92 billion which is 29% of the total agricultural gross value of R312 billion gross value [1]. Thus, the informal markets, such as fresh fruits and vegetables produce markets, are responsible for the highest distribution of fresh produce. The viability and sustainability of informal vegetable markets will exist as long as there is substantial demand from consumers in urban areas [2]. Farm activities can influence the progression of pathogens on the commodity, including market microbial hazards.

In various parts of South Africa vegetables are being widely cultivated traditionally by rural farmers for several decades. The farmers provide vegetables to street vendors, local restaurants including local markets. Washing and disinfecting is part of minimal processing to inactivate pathogenic microorganisms that may be present by rinsing away exudates that would otherwise provide nutrients for microbes, thereby reducing microbial load. Due to favourable conditions and certain survival strategies, pathogenic microbes are well able to thrive and shift to a new niche and form microbiota [3]. The emergence and proliferation of dominant microbial populations are caused by the wide range of metabolic mechanisms found in the microbial pool [4]. Studies have highlighted the advantages and disadvantages of washing fresh perishable vegetables with various disinfectants, including their limitations and alternatives [5, 6]. For example, other studies showed how the depletion of free-chlorine disinfectants attributed to the dissemination of contamination of pathogens between different fresh vegetables and fruit commodity batches [7, 8]. [9] demonstrated that failure to maintain disinfectant levels in the washing of spinach resulted in cross-contamination between the batches leading to an outbreak with 200 confirmed cases of food poisoning and seven deaths. Studies by Abatcha et al. [10] and Yang et al. [11] also highlighted the statistics of foodborne poisoning cases including deaths caused by foodborne pathogens isolated from contaminated leafy greens.

The interaction that occurs between pathogens and fresh vegetables is multifaceted with the probability of contamination occurring at critical points such as harvesting, initial processing at the facility, transportation, and final preparation in the kitchen [12]. This is one of the critical reasons to conduct comprehensive research on the interaction, microbial phyllosphere and microbial traits, including the transition, antagonist behaviour, resources, and dominance of these microbes on plants. Hazard analysis critical and control points including food safety programmes are imperative to avoid any available hazards during production and cold chain. The study is important as it identifies what human being consumes as these commodities are consumed raw and as part of a nutritional diet and staple food which is mostly consumed in South Africa.

## Materials and methods

### Study area

**Fig. 1:**
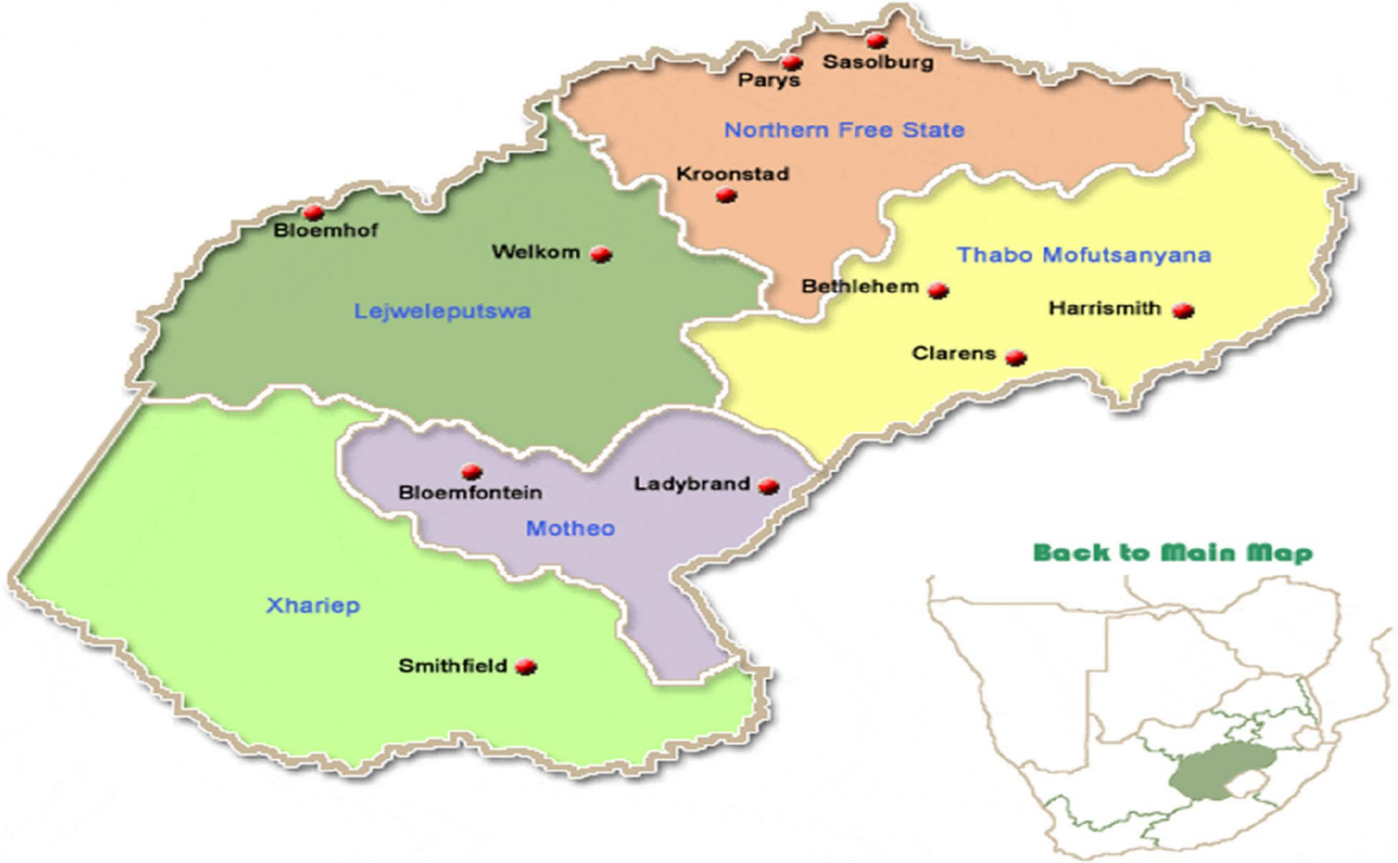
South Africa map showing free state province where the samples were collected

The present study was conducted by procuring two hundred and fifty samples of raw unpackaged spinach phyllosphere and hundred and ninety samples of cabbage heads from five markets that are mostly supplied by small-scale farms (Fig. 1). The selected markets represent the major markets that supply most leafy greens to consumers, making the results of the study representative. Samples were collected in the following towns in the Free State Province, South Africa: Motheo District - Bloemfontein (29.1217°S, 26.2128°E), Lejweleputwa District - Henneman (28.9784°S, 27.0264°E), Thabo Mofutsanyana District - Ladybrand (28.5225°S, 27.5241° E, Fezile Dabi District - Kroonstad (27.6373°S, 27.2323°E), and Thabo Mofutsanyana District - Ficksburg (29.1136°S, 27.2718°E). The province is located on the highveld, a plateau rising to elevations of 6,000 feet (1,800 m) in the east and sloping to about 4,000 feet (1,200 m) in the west. The climate varies from warm and temperate with an annual rainfall of 40 inches (1,020 mm) in the east to semiarid with rainfall of only 15 inches (380 mm) in the far west. Mean annual surface temperatures gradually increase from about 58° F (14° C) in the east to 62° F (17° C) in the west. Frost and rainfall are common over the entire province.

### Sampling technique

The study design used random sampling which was conducted on spinach and cabbage samples from three different sections of the stored samples ready for purchase. To ensure that sample collection was representative, at least six areas, the middle part and two sides of the stored samples were assessed. The samples collected were selected based on the sampling technique in which each sample has an equal probability of being chosen. A sample chosen is meant to be an unbiased representation of the total population [13]. Leafy green samples were analysed for each of the following microorganisms or microbial species: total aerobic mesophilic bacteria, Enterobcateriacea (total coliforms), coagulase-positive Bacillus and Listeria. All samples were collected aseptically, subsequently transported to the laboratory, and were prepared and plated on various pre-solidified agars from the samples homogenates and incubated within 12 h, on the same day of collection. Duplicates of aerobic mesophilic count, Enterobacteriaceae count (total coliform), Bacillus count, and Listeria count were enumerated from the homogenate of the samples prepared. Plate count agar, including selective media such as Violet red bile agar (VRBA), Bacillus chromoSelect agar (Merck, South Africa), and Brilliance chromogenic Listeria [ThermoFisher, Scientific, South Africa] was utilised [14]. The isolated colonies were counted using an 80 Scan 1200® Automated Colony Counter [Interscience]. The mean number of colonies counted for all count types was expressed in log colony forming units (CFUs).

### Total aerobic mesophilic and Enterobacteriaceae

The enumeration of the total viable aerobic mesophilic count was determined utilising plate count agar (PCA) and nutrient agar medium. Samples were serially diluted in buffered peptone water (BPW), whereafter aliquots of 0.1 ml were inoculated using the plate count spread-plate technique, and incubated at 37ºC/48 h [14]. To count, 0.1 ml of a10^1^–10^5^ serial dilution of the leafy green vegetable samples was spread plated on violet-red bile agar and Plate count agar. Plates were incubated at 32 °C for 24 h after spreading. Red to pink colonies, surrounded by precipitated bile, were counted as coliforms [15].

### Isolation of Bacillus spp

Samples were serially diluted in BPW, inoculated on Bacillus ChromoSelect agar, and spread evenly onto the surface of each plate with a sterile glass spreading rod before being incubated at 37ºC for 48 h [16]. Peacock blue colonies with blue zones were subjected to appropriate biochemical tests such as basic characterization which include gram staining, motility, fermentation and enzymatic reaction were observed [17, 18].

### Isolation of Listeria spp

Approximately 25 g of each sample was homogenized with Listeria broth and stomach for a minimum of 30 s to thoroughly mix the sample. The broth was incubated without agitation at 30ºC for 24 ± 2 h. The bag was gently agitated and a microbiological loop was utilised to remove 0.1 ml and inoculate it onto a Brilliance Listeria agar plate (chromogenic) accodign to Mritunjay and Kumar, 2015 [19]. The inoculum was carefully spread as soon as possible over the surface of the plate using a sterile spreader without touching the sides of the plate with the spread. The inoculated plates were inverted and incubated at 37ºC for 24 ± 2 h. The plates were examined for blue colonies with and without opaque white halos [ISO 16140 standard]. As an additional test, the type of haemolysis was observed [20].

### Biochemical tests

Isolates were further characterized biochemically using API 20E for Enterobacteriaceae and related genera whilst API 20NE was utilised for the identification of non-fastidious and non-enteric Gram-negative rods. API 50 CHB/E Medium was utilised for the identification of Bacillus and related genera, as well as Gram-negative rods belonging to the Enterobacteriaceae and Vibrionaceae families and API Listeria for the identification of Listeria [bioMérieux, France]. The tests were performed according to the manufacturer’s instructions.

Colonies were streaked out on plate count agar plates and blood agar for pure colonies. Briefly, 1–4 colonies of identical morphology from young cultures (18–24 h) were picked and emulsified in 5 ml of sterile sodium chloride (0.85%) for API 20E, 20NE, and 50CHB/E and the turbidity was adjusted to the equivalent of the turbidity of 0.5 McFarland standards. The standardized bacterial suspension was carefully distributed into the tubes of the test strip to avoid the formation of bubbles. Anaerobiosis was created by overlaying with sterile mineral oil and the strips were subsequently incubated in a humid atmosphere for 18–24 h at 37 °C.

### Data analysis

Data interpretation was performed using the API database with the apiwebTM identification software to obtain the identification result for each strain tested. Statistical analysis of the data on growth medium influencing the prevalence of pathogens and microbial mean counts was performed using the general linear model of SAS software version 9.2 to determine the analysis of variance (ANOVA). Tukey’s least significant difference (LSD_T_), described by Steel and Tourie (1980), was utilised to determine the significant results between variants. The statistical difference between treatment means was determined at the (p ≤ 0.05) probability level. The Shapiro-Wilks test was performed on standardised residuals to test for any deviations from normality [21]. The growth medium influencing the prevalence of pathogens and the microbial mean count was subjected to multivariate data analysis, using principal component analysis (PC-XLSTAT 2015) to identify and evaluate the groupings between the variables.

## Results and discussion

### Cabbage phyllosphere microbial count concentrations

A significant interaction between markets in different towns and microbial mean counts in 10^3^ and 10^4^ concentrations was observed (Table 1). Microbial concentrations 10^3^ and 10^4^ had highly significant (P < 0.05) microbial mean counts. The h microbial mean count observed for PCA in 10^4^ in Ladybrand Ficksburg, Kroonstad correlated followed by Bloemfontein the lowest count in Hennemann, respectively.

**Table 1:** Mean log_10_ cfu/ml of bacteria sampled from cabbage phyllosphere from different markets

Factors	10^1^	10^2^ Cons	10^3^ Cons	10^4^ Cons
Markets				
Bloemfontein	69.83 ± 37.08^b^	53.00 ± 34.64^a^	36.16 ± 28.39^a^	46.00 ± 20.42^a^
Ficksburg	82.50 ± 31.04^a^	54.16 ± 24.29^a^	33.00 ± 10.84^a^	22.33 ± 11.48^c^
Hennemann	67.50 ± 48.16^b^	64.66 ± 65.10^a^	39.00 ± 38.39^a^	39.33 ± 11.15^a,b^
Kroonstad	72.00 ± 38.63^b^	57.50 ± 32.98^a^	32.50 ± 6.83^a^	14.00 ± 8.24^c^
Ladybrand	85.33 ± 14.73^a^	58.33 ± 31.75^a^	37.66 ± 18.28^a^	27.83 ± 24.14^b,c^
*F*-value	8.76	2.05	0.38	7.67
*P*-value	0.0026	0.1796	0.0205^*^	0.0153
Agar				
Bacillus	45.33 ± 18.92^b^	26.60 ± 8.13^b^	19.40 ± 10.09^b^	10.66 ± 3.84^b^
PCA	105.53 ± 9.15^a^	90.46 ± 26.93^a^	51.93 ± 18.44^a^	36.33 ± 16.60^a^
*F*-value	624.83	109.37	61.28	23.65
*P*-value	0.0001	0.0001	0.0001	0.0028
Markets x Agar				
*F*-value	19.90	3.14	7.14	5.87
*P*-value	0.0001	0.0649	0.0055^*^	0.0386^*^

^*^significant

As illustrated in Table 1, a significantly (P < 0.05) high microbial mean count for 10^3^ and 10^4^ were observed for PCA in all markets. The microbial mean count observed for PCA in Ladybrand, Ficksburg, and Kroonstad correlates compared to Bloemfontein and Ladybrand microbial counts. Ficksburg and Ladybrand microbial mean count for Bacillus in all markets correlates.

**Fig. 2:**
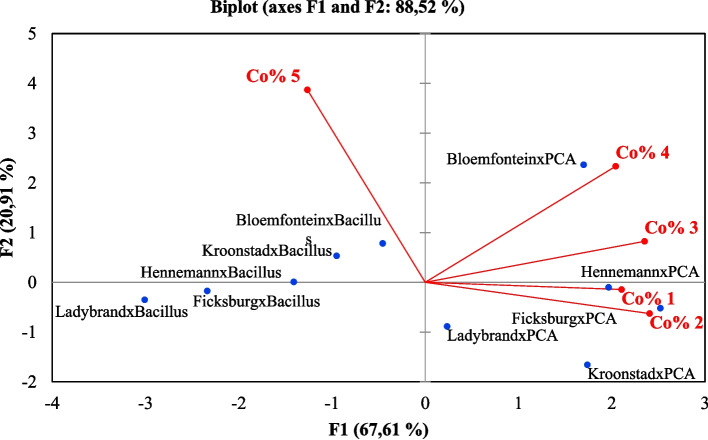
Principal component biplot illustrating the variations of cabbage microbial mean count correlation in different concentrations from farms in different growth media. Abbreviations: PCA = Plate count agar

Multivariate data analysis was applied using a PC analysis to group correlating microbial mean counts. Results from this method were comparable to those of an ANOVA (Fig. 2).

The score plot and loading matrix, based on the first and second principal components (PC1 and PC2) accounted for 92.74% of the total variance. The biplot loading in PC 1 (64.70) showed that the microbial mean counts for PC analysis from Ladybrand and Bloemfontein correlated with concentration percentages 4 and 5. Ficksburg, Ladybrand and Kroonstad microbial mean counts correlated with concentration percentages 1, 2 and 3. Concentration 10^3^ and 10^4^ were considered significant to the (*P* < 0.05) compared to concentration 10^1^ and 10^2^.

### Spinach phyllosphere microbial count concentrations (Analysis of Variance)

Significant interactions between market and concentrations were observed on spinach phyllosphere samples with concentrations 10^4^ (Table 2).

**Table 2:** Mean log_10_ cfu/ml of bacteria sampled from spinach phyllosphere from markets

Factors	10^1^ Cons	10^2^ Cons	10^3^ Cons	10^4^ Cons
markets				
Bloemfontein	86.66 ± 59.42^c^	78.66 ± 16.07^a^	67.00 ± 12.28^a^	43.00 ± 10.53^a^
Ficksburg	80.00 ± 40.65^a,b^	69.50 ± 38.11^a^	44.66 ± 22.88^b^	27.83 ± 13.04^b^
Hennemann	86.66 ± 37.21^a^	66.50 ± 33.58^a^	45.00 ± 12.99^b^	28.50 ± 10.27^b^
Kroonstad	78.83 ± 28.35^a,b^	72.50 ± 39.09^a^	50.00 ± 20.42^b^	23.33 ± 7.52^b,c^
Ladybrand	72.33 ± 36.95^b^	41.16 ± 21.14^b^	27.83 ± 13.93^c^	14.6 ± 12.06^c^
*F*-value	16.73	7.27	6.48	10.01
*P*-value	0.0002	0.0090	0.0125	0.0033^*^
Agar				
Bacillus	38.40 ± 19.19^b^	33.66 ± 8.38^b^	28.83 ± 9.07^b^	16.83 ± 9.37^b^
PCA	111.40 ± 10.94^a^	88.66 ± 22.01^a^	57.33 ± 16.62^a^	32.86 ± 10.90^a^
*F*-value	858.90	130.40	31.42	25.21
*P*-value	0.0001	0.0001	0.0005	0.0010
markets x Agar				
*F*-value	13.62	2.53	0.52	3.59
*P*-value	0.0005	0.1303	0.6811	0.0657

^*^_significant_

The highest microbial mean count difference for 10^4^ was observed in Hennemann followed by Ficksburg, Bloemfontein and Ladybrand, with the lowest counts observed in Kroonstad, respectively. The highest microbial mean count for Bacillus was observed in Henneman with the lowest count observed in Bloemfontein, respectively. *Microorganisms identified from spinach and cabbage contamination from markets*. Table 3, 4, 5, 6 and 7 demonstrates the pathogens isolated, their microbial counts including dominant opportunistic pathogens identified in both spinach and cabbage.

**Table 3:** Microbial loads present in spinach and cabbage from various markets

**Microbial**	**Samples**	**Log CFU/g**				**Total (%)**
		1–1.9	2–2.9	3–3.9	4–4.9	
AMB	Spinach	-	52/52 C. i	84/84 A. h 27/63 B. c	69/69 P. a 36/63 B. c	268(71.7%)
	Cabbage	-	51/51 A. h 33/67 B. c	53/53 P. a 34/67 B. c	-	171(45.7%)
ENTB	Spinach	33/97 E. c 62/62 E. a	67/67 E.cc 46/88 P. m	29/88 P. m 30/97 E. c	13/88 P. m 34/97 E. c	314(84%)
	Cabbage	-	21/47 E.cc	21/65 E. c 26/47 E.cc	44/65 E. c 49/49 P. m	161(43.0%)
Bacillus	Spinach	-	4/51 Bac	47/51 Bac	-	51(13.6%)
	Cabbage	-	42/42 Bac	-	-	42(11.2%)
Listeria	Spinach	12/31L. m 21/34 L. i	19/31L. m 13/34 L. i	-	-	65(17.4%)
	Cabbage	-	-	-	-	-

*AMB* aerophilic mesophilic, *ENTB* Enterobacteriaceae, *C. i* C. indologenes; A. h—A. haemolyticus; P. a—P. aeruginosa; B. c—B. cepacia; E. c- E. coli; E. cc—E. cloacae; P. m—P. mirabilis; Bac- Bacillus; L. m—L. monocytogenes; L. i – L. ivanovii

**Table 4:** Mean log_10_ cfu/ml of bacteria isolated from cabbage phyllosphere from different markets

**Isolates**	**Markets**	**Pathogens identified**
Cabbage phyllosphere	Bloemfontein	Acinetobacter haemolyticus, Escherichia.coli (99.9%), Pseudomona luteola
	Kroonstad	Pseudomona aeruginosa, Brevibacillus laterosporus, Bacillus subtilis, Yersinia enterolitica, Enterobacter aerogenes
	Henneman	Burkholderia cepacia (99.7%), Proteus mirabilis(99.9%), Enterobacter cloacae (97.7%), next taxon Enterobacter amnigenus with (1.5%), Pseudomonas oryhabitants, Enterobacter aerogenes (98.8%), Acinetobacter lwoffii, Escherichia coli (99.9%), Chryseomonas luteola
	Ficksburg	Bacillus subtilis, Escherichia coli, Providencia alcalifaciens
	Ladybrand	Burkholderia cepacia, Bacillus cereus, Bacillus subtilis, Burkholderia gladioli

(%) – viable species count shown by API Web

**Table 5:** Depicts the number of isolated species and percentage of positive species from cabbage samples

Leafy green type (total no. of isolated species)	Number of isolated positive species from collected samples (% of positive species)						
	A. haemolyticus	P. aeruginosa	B. cereus	E. cloacae	B. cepacia	P mirabilis	E.coli
Cabbage (*n* = 374)	51 (13.6%)	53 (14.1%)	42 (11.2%)	47 (12.6%)	67 (18%)	49 (13.1%)	65 (17.4%)

**Table 6:** Pathogens identified from spinach phyllosphere isolates

Spinach phyllosphere	Bloemfontein	Chryseobacterium indologenes (99.7%), Escherichia coli, Proteus penneri, Pseudomona luteola, Acinetobacter haemolyticus
	Kroonstad	Bacillus cereus, Escherichia coli, Bacillus lentus (98.5%), and Bacillus subtilis (0.8), Enterobacter cloacae (99.3%) next taxon Enterobacter asburiae with (0.2%), Bacillus megaterium, Listeria spp. (Listeria ivanovii and Listeria monocytogenes)
	Henneman	Pseudomonas putida, Bacillus megaterium, Enterobacter aerogenes (98.8%), Escherichia coli (99.9%), Brevibacillus laterosporus (97.7%), next taxon Bacillus pumilus (0.8%), Bacillus cereus, Bacillus lentus (98.5), Proteus mirabilis (99.8%), next taxon Proteus Vulgaris group (0.2%), Chryseomonas luteola, Listeria ivanovii (96.4%), Listeria monocytogenes (99.9%)
	Ficksburg Ladybrand	Brevibacillus non-reactive, Pseudomona luteola Burkholderia cepacia, Pseudomanas stutzeri, Proteus mirabilis, Pseudomona aeruginosa Pseudomona luteola, Escherichia coli, Proteus mirabilis, Burkholderia cepacia (ficksburg and Ladybrand are two different towns, so they need to be separated)

(%) – viable species count shown by API Web

**Table 7:** Depicts the number of isolated species and percentage of positive species from spinach samples

Leafy green type (no. of isolated species	Number of isolated positive species from collected samples (% of positive species)					
	C. indologenes	P. mirabilis	L. monocytogenes	L. ivanovii	E. asburiae	E.coli
Spinach (*n* = 698)	52 (7.4%)	88 (13%)	31 (4.4%)	34 (4.9%)	62 (8.9%)	97 (13.9%)
	A. haemolyticus	P. aeruginosa	B. cereus	E. cloacae	B. cepacia	
	84 (12%)	69 (9.9%)	51 (7.3)	67 (9.6%)	63 (9.1%)	

### Multivariate data analysis

**Fig. 3:**
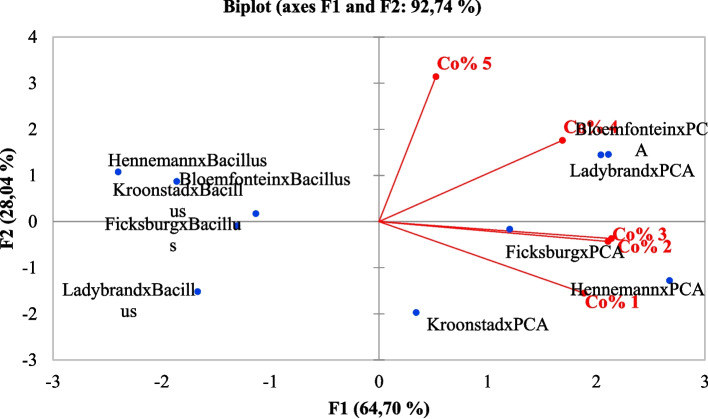
Principal component biplot illustrating the variations of spinach microbial mean count correlations in different concentrations in different farms using different growth media

Multivariate data analysis was applied using a PC analysis to group correlating microbial mean counts. Similar results to the ANOVA were obtained using this method (Fig. 3). Figure 3  below depicts the variations of spinach mean counts and their correlation in different concentrations. Different markets were compartmentalised according to their concentrations in various agars. The score plot and loading matrix, based on the first and second principal components (PC1 and PC2), accounted for 88.52% of the total variance. The biplot loading in PC 1 (67.61%) showed that the microbial mean count for PC analysis correlates with concentrations 1, 2 and 3. Henneman, Ficksburg and Kroonstad microbial mean counts correlated. Concentration 10^4^ was considered significant to the (*P* < 0.05) compared to concentration 10^1^ 10^2^ and 10^3^.

### Microorganisms identified from spinach and cabbage contamination from markets

The figure below summarises the microbial count for both spinach and cabbage samples collected from various markets.

**Fig. 4:**
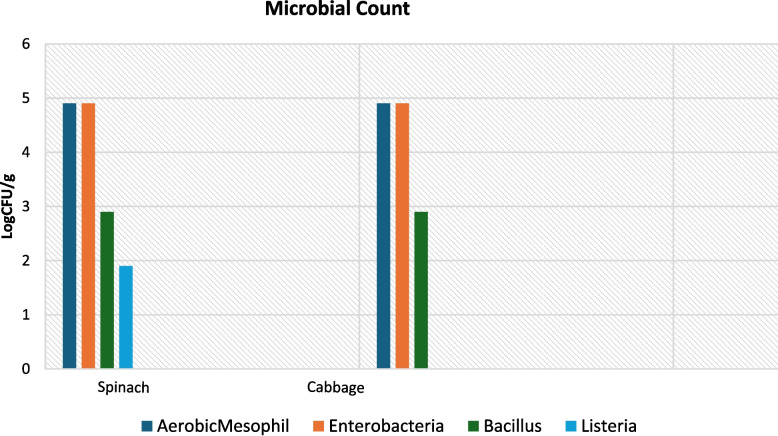
Microbiological count of leafy green vegetables collected from markets

Among leafy green samples enumerated, 17.4% and 13.9% were contaminated by E. coli which is highly prevalent in cabbage and spinach followed 13.1% and 13% P. mirabilis and 12.65 and 9.6% C. cloacae including 4.4% of L. monocytogenes and 4.9% L. ivanovii in spinach. Listeria species was not detected in any cabbage samples. The highest count was Enterobacteriaceae ranged from 1–4.9 log CFU/g (84%), aerobic mesophilic bacteria ranged from 2–4.9 log CFU/g (71.7%) in spinach followed by mesophilic with 2–3.9 log CFU/g (45.7%) and Enterobacteriaceae range from 2–4.9 log CFU/g (43%) and aerobic while the minimum was in spinach samples 1–2.9 log CFU/g for Listeria species. Al-Holy et al. [22] reported that the counts for Enterobacteriaceae ranged from 5.72 to 7.06 log10 cfu/g in lettuce and leek, respectively while high total coliform counts were also detected, where counts ranged from 5.86 to 6.59 log10 cfu/g in the tested fresh leafy green vegetables sold from local markets (Fig. 4).

A study in South Africa reported that the mean coliform count on spinach 4.78 log CFU/g was significantly higher than that on cabbage which was 3.65 log CFU/g, highlighting that both cabbage and spinach from retails were not significantly different [23]. Degaga et al. [24] also reported the maximal count for aerobic mesophilic bacteria enumerated in cabbage to be 6.4 log CFU/g while the maximum Enterobacteriaceae count were recorded in cabbage was 5.7 log CFU/g. Foods containing aerobic mesophilic bacteria are considered as good (< 4 log10 CFU/g), average (4.0–6.7log10 CFU/g), poor (6.7–7.7 log10 CFU/g), and spoiled food (> 7.7log10 CFU/g) [25].

### Cabbage phyllosphere pathogens

The table below depicts pathogens enumerated from cabbage samples, the viable species count is shown in percentages next to each species.

The table below shows the total number of isolated species from cabbage and percentage of enumerated pathogens from the commodity. The percentage of enumerated pathogen is also provided next to the total number.

Several studies have reported microbiological quality of leafy green from markets. Acinetobacter spp. are aerobic gram-negative organisms and are known skin colonisers seen as normal flora of the oropharynx and skin in approximately 25% of healthy individuals. They have been identified as a cause of nosocomial infections like septicaemia, pneumonia, meningitis, urinary tract, skin and wound infections [26]. In this study,

Acinetobacter haemolyticus (A. haemolyticus) ranged from 3–3.9 log CFU/g in spinach and 2–2.9 log CFU/g in cabbage. It is assumed that contamination of was from agronomic activities, owing to its capacity to survive long periods on dry surfaces and persist in the environment. A study from Portugal, Carvalheira et al. [27] reported that Acinetobacter prevalence detected on lettuce (Lactuca sativa) from food market was 86.7% and 29.8% while 4.4% of the strains were classified as extensively drug-resistant. Good hygiene and handling practices associated with food processing as well as disinfection are very important to avoid and mitigate contamination. Pseudomonas spp. emanates from soil and water environments, particularly those associated with human activity [28]. Additionally, Pseudomonas aeruginosa (P. aeruginosa) is considered an opportunistic pathogen due to its wide range of infections and is often hard to treat. In this study, P. aeruginosa ranged between 4–4.9 log CFU/g in spinach and 3–3.9 log CFU/g in cabbage. In Spain, Söderqvist et al. [29] reported that the composition of baby spinach and mixed-ingredient salad bacterial communities changed during cold storage (8 °C), with Pseudomonas being the most abundant high-level taxonomic group across the samples. Khiyami et al. [30] reported that AMB counts were between 5 log CFU/g and 5.7 log CFU/g for cabbage while Büyükünal et al. [31] reported lettuces and spinach were also positive with 3.6 log10 CFU/g for AMB. It is assumed that P. aeruginosa and P. luteola presence is due to insufficient hygiene during the processing and the presence of N-acyl homoserine lactones (AHL) which is responsible for biofilm formation and virulence. Highly damaged leaves which are caused by processing should be removed before packaging as exudes attracts opportunistic pathogens. These pathogens are well able to grow in storage temperature indicating contamination due to conducive environment. The habitat of Brevibacillus overlaps with Bacillus and they are widespread genera of gram-positive bacteria, recorded from diverse environmental habitats, including soil [32]. Bacillus cereus (B. cereus) belongs to the same subgroup as Bacillus subtilis (B. subtilis), by both phenotypic and rRNA sequence classification. Bacillus species are a common cause of foodborne illnesses in humans, and one of its most distinct features and survival mode is the ability to produce heat-resistant spores. Several studies also reported on toxigenic diversity and cytotoxicity of the Bacillus spp. isolated from fresh produce and the effects of various factors on the growth of B. cereus [33, 34]. In this study, B. cereus highest count ranged from 2–3.9 log CFU/g in spinach and 2–2.9 log CFU/g in cabbage. Contamination from Bacillus spp. may be due to its ability to withstand certain conditions and thrive, forming part of the bacterial composition. Kim et al. [35] reported the occurrence rate of B. cereus and contamination level in organic vegetables on sale in retail markets and highlighted that the mean contamination level to be significantly higher 1.86 log CFU/g vs. 0.69 log CFU/g (p < 0.05). In addition, six samples of organic vegetables were found to be contaminated with B. cereus at over 4 log CFU/g which is categorized as unsatisfactory according to Health Protection Agency guideline.

Enterobacter species are ubiquitous and originate from terrestrial and aquatic environments such as water, sewage, soil, and food including the intestinal tract of humans, can be present in human skin surfaces causing Urinary tract and other infections. Enterobacter cloacae (E. cloacae) is an opportunistic pathogen due to contamination from handling and inadequate sanitation. A study [36] found Enterobacter to be the dominating genus that was associated with leafy salad vegetables. On this study, the highest population count for spinach ranged from 2–2.9 CFU/g and 2–3.9 CFU/g for cabbage. It is identified as a contaminant on the farm with a possible succession to the market, thus it is an indicator of cross-contamination. A study reported that Iceberg lettuce samples had a low contamination by coliforms with 1.44 and 1.23 log10 cfu g-^1^, while high populations were found in rocket and spinach, 94 and 93% with 3.44 and 3.41 log10 cfu g-^1^, respectively [37]. In South Africa, Enterobacter, Serratia, E.coli was reported to be observed in irrigation water including leafy green while Proteus spp. was observed in irrigation water only in farming site E [38]. Mustafa et al. [39] identified T1SS, T2SS, T4SS and T6SS in five E. cloacae complex strains highlighting that the T3SS, which is a key component in the bacterial structure helps gram-negative pathogens invade the host with an exclusive mechanism of virulence and enables them to bypass the extracellular barriers.

Burkholderia species are opportunistic pathogens found on and are reported to be pathogens in leafy green vegetables. These beneficial Burkholderia species are free-living or endophytic and form mutualistic associations with their host plants [40]. In this study, Burkholderia cepacia (B. cepacia) AMB ranged from 3–4.9 log CFU/g in spinach and 2–3.9 log CFU/g in cabbage. This endophytic bacterium could be part of plant-promoting endophytic bacteria having a mutual relationship with other opportunistic bacteria available in the cabbage phyllosphere or contamination from agricultural soil. Few studies have confirmed Burkholderia are widely distributed in agricultural soils including two celery samples [41]. Harrelson et al. [42], a recent study which identified B. cepacia leafy green vegetables such as kale (Brassica oleracea and Brassica napus), cabbage (Brassica rapa subsp. Pekinensis), lettuce and chard (Beta vulgaris) varieties. To date, there has been limited enumeration of B. cepacia prevalence in leafy green vegetables compared to investigations of their clinical epidemiology.

### Spinach phyllosphere pathogens

Tabulation of various opportunistic pathogens enumerated from spinach samples collected from various markets.

The table below shows the total number of isolated species from spinach and percentage of enumerated pathogens from the commodity. The percentage of enumerated pathogen is also provided next to the total number.

Chryseobacterium indologenes (C. indologenes) emanates from soil, water and food products, [43]. In this study, C. indologenes ranged from 2–2.9 log CFU/g (100%) in spinach, none was detected in cabbage. It is perceived as part of microbial population as a coloniser, that is resistant to disinfectant and a food spoilage organism. Other literature suggests that C. indologenes spp. can resist chlorination, specifically at water municipal plants, which can indicate possible water contamination from the sewer [44]. Future research on this subject can be useful to identify all the pathogens resistant determinants to water chlorination and develop countermeasures.

Proteus mirabilis (P. mirabilis) is ubiquitous in the environment and is regarded as a part of the normal flora in the human gastrointestinal tract. In this study, ranged from 2–4.9 log CFU/g in spinach and 4–4.9 log CFU/g (100%) from cabbage. P. mirabilis could be attributed to faecal contamination due poor sanitation and to poor hygiene or by non-composted or improperly composted manure utilised as fertiliser on spinach. Imoni et al. [45] reported the prevalence of Enterobacteriaceae in fresh leafy vegetables obtained from Benin, Nigeria and observed that P. mirabilis had the lowest total Enterobacteriaceae counts of 6.42% and E. aerogenes with 22.94%. Among the foodborne disease incidents reported in various countries, the proportion of food poisoning caused by P. mirabilis remains high [46]. For example, from 1998 to 2013, it was reported that 294 people had food poisoning caused by P. mirabilis in 3 provinces of China (Gong et al. [47]). Yaseen et al. [48] also reported that adhesion genes like mrpA and ZapA play a crucial role in facilitating attachment, biofilm formation including immune evasion and that the mrpA gene is integral to the pathogenesis of P. mirabilis. In another study, Liu et al. [41] highlighted that whole-genome analysis of bla_ndm_-bearing proteus mirabilis isolates carrying bla_ndm_ from lettuce vegetables in China suggest that sustained surveillance of these foodborne pathogens among fresh vegetables is urgent to ensure the health of food consumers. However, reports, especially about P. mirabilis on fresh vegetables, are still lacking [41].

In this study, L. monocytogenes ranged between 1–2.9 log CFU/g in spinach only. Owing to the Listeria spp. capabilities, it was assumed that it might emanate from a farm and form biofilm as a strategy to survive harsh and stressful environments. Another possibility could be the temperature fluctuation of spinach during distribution and poor sanitary practices of workers which might have proliferated the pathogen. Tango et al. [49] indicated the prevalence of Listeria monocytogenes (L. monocytogenes) to be highest on organic romaine lettuce and spinach and found 4 of 63 (6.4%) samples of each type of vegetable with the majority of deaths amounting to thirty-three from a single outbreak. Listeria monocytogenes was prevalent in 5 out of 10 coriander leaves, 2 out of 4 spinach samples and one from 4 cabbage samples from a local market ranged from < 3 to > 1100 whereas for ready-to-eat salads it was 11 to 460. Furthermore, the study concluded that evidence of higher number of coliforms was observed mostly in green leafy vegetables [50]. Harter et al. [51] mentioned that L. monocytogenes type ST121 stress survival islet (SSI-2) together with the transcriptional regulator, σ^B^ plays a role in the survival of the cell during detergent stresses at lethal levels in survival under oxidative and alkaline stresses response and activation of biofilms in the food processing environment. Yoon et al. [52] reported that the L. monocytogenes biofilm formation is the cause of increment to quaternary ammonium compounds tolerance by increasing membrane hydrophobicity promoting further adherence to objects or surfaces.

Previous studies reported that Listeria ivanovii (L. ivanovii) was exclusively linked to ruminants only, but it was later highlighted that L. ivanovii infections occurred in humans after the ingestion of contaminated food [53]. In another study, Nyenje et al. [54], a total of 51 L. ivanovii strains were isolated from ready-to-eat including vegetables purchased from cafeterias in Alice, South Africa. In this study, L. ivanovii ranged from 1–2.9 log CFU/g in spinach indicating possible contamination from farms with livestock production, including inadequate sanitation and poor hygiene. The primary implication of L.ivanovii might be greater than currently documented as it is overshadowed by L. monocytogenes owing to its sporadic case compared to the two pathogens. Another distinguishable reason contributing to under-document is lack of severe symptoms which may be clinically less recognizable, its lower virulence compared to L. monocytogenes means in a healthy person its asymptomatic resulting in undiagnosed and unreported. There is less information available in the literature on the prevalence and distribution of L. ivanovii along the food chain, although it appears that, apart from L. monocytogenes, L. ivanovii is the most frequently isolated Listeria species.

Enterobacter asburiae (E. asburiae) emanates from soil, water and food products and is also known as the epiphytic bacterium [55]. In this study, E. asburiae ranged from 1–1.9 log CFU/g (100%) from spinach. It is speculated to be a pathogen, although more studies need to be carried out to explore the mechanisms involved in the pathogenesis. Lau et al. [56] shared light on E. asburiae isolated from lettuce ability to produce AHL which is for the regulation of virulence and biofilm formation including biocidal resistance. However, further studies are required to verify the presence and function of virulence-related genes and complex proteins produced by E. asburiae and their regulation in interspecies microbial growth suppression [55]. Spahr, Endimiani and Perreten, [57] reported that the strain harboured a 163-kb conjugative IncFII (Yp) plasmid containing blaIMI − 6 and putative virulence genes genetically related to strains from a patient in France and from retail salad in Switzerland. The finding suggests possible trans-sectoral dissemination of IMI-producing bacteria raising concerns since they could further spread into the community. To date, reports or studies of E. asburiae on fresh produce are still very limited.

Escherichia coli (E. coli), is a type of bacteria that normally lives in humans and animals. It is often used as an indicator for poor hygiene and sanitation in food processing environments. It is highly virulent, with a low infectious dose (10 to 100 CFU) and approximately 100,000,000 organisms must be ingested to cause illness in a healthy person. In this study, E. coli ranged from 3–4.9 log CFU/g in both spinach and cabbage. It is assumed to be prevalent due to unsanitary practises which might emanate from poor hygiene and poor post-harvest disinfectant. Another study reported that contamination at high levels ranging from 6 and 7 log CFU/g allowed E. coli O157:H7 to spread over all processed lettuce samples, resulting in detectable concentration values in 100% analysed samples [58].

## Conclusion

This study provides data regarding pathogen prevalence in farms, microbial quality and succession to markets highlighting poor surveillance and monitoring which appears to be increasing and spreading. The data provided is expected to support hazard analysis and epidemiological surveillance on the prevalence of pathogens from leafy greens. Understanding the complex microbiome ecosystem that is unique for each product is imperative as it provides directives on mitigation and prevention of outbreak infections including control of the pathogen during outbreak or recall. Good hygiene practices must be applied until the purchase stage to ensure food safety. The study contributes to the scholarly research on the primary production and markets food safety of fresh leafy green. It also contributes to the understanding of contamination and potential risk parameters that could influence the predominance of leafy green vegetables. The data may be utilised to influence further educational efforts and future research gaps designed to provide risk mitigation for small-scale Free State farms and markets regarding spinach and cabbage production. Insufficient knowledge is available on leafy green vegetable production in the Free State except for their nutrition contents as these are staple foods.

## Supplementary Information

Supplementary Material 1.

## Data Availability

All data generated or analysed during this study are included in this published article.

## Abbreviations

A. haemolyticus: Acinetobacter Haemolyticus
AMB: Aerophilic mesophilic
ANOVA: Analysis of variance
API: Analytical profile index
B. cepacia: Burkholderia cepacia
BPW: Buffered peptone water
C. indologenes: Chryseobacterium indologenes
CFU/g: Colony forming units per gram
E. coli: Escherichia coli
E. cloacae: Enterobacter cloacae
ENTB: Enterobacteriaceae
L. ivanovii: Listeria ivanovii
L. monocytogenes: Listeria monocytogenes
P. aeruginosa: Pseudomona aeruginosa
P. mirabilis: Proteus mirabilis
PCA: Plate count agar
PC: Principal component analysis
LSD_T_: Tukey’s least significant difference
VRBA: Violet red bile agar
