# Supplementary material for: Succession and proliferation of opportunistic pathogens on leafy greens at retail markets
Source: BMC Microbiol. 2026 Mar 7;26:351. doi: 10.1186/s12866-026-04823-0 (PMC13081453; doi:10.1186/s12866-026-04823-0)
Supplement: Supplementary file 1 — Supplementary Material 1. [file 12866_2026_4823_MOESM1_ESM.docx]

A significant interaction between retails in different towns and microbial mean counts in different growth media was observed in all concentrations except for 10^2^ concentrations (Figure 4.4.2). Microbial concentrations 10^1^, 10^3^ and 10^4^ had highly significant value of (*P*<0.05) microbial mean counts.

**Figure A:** Microbial mean counts for cabbage in this study are presented graphically as mean log_10_ cfu/ml (concentration 10^1^).

**_Abbreviations_**_: Asterisk (*) = Interactions of interest for discussion._

**Figure B:** Microbial mean counts for cabbage in this study are presented graphically as mean log_10_ cfu/ml (concentration 10^3^).

**_Abbreviations_**_: Asterisk (*) = Interactions of interest for discussion._

**Figure C:** Microbial mean counts for cabbage in this study are presented graphically as mean log_10_ cfu/ml (concentration 10^4^).

**_Abbreviations_**_: Asterisk (*) = Interactions of interest for discussion._

Figure below 4.4.3 Significant interactions between markets and concentrations were observed on spinach phyllosphere samples with concentrations 10^1^

**Figure A:** Microbial mean counts for spinach in this study are presented graphically as mean log_10_ cfu/ml (concentration 10^1^) .

**_Abbreviations_**_: Asterisk (*) = Interactions of interest for discussion_
